# Supplementary figures and images for: Data-driven predictive modeling for massive intraoperative blood loss during living donor liver transplantation: Integrating machine learning techniques
Source: PLoS One. 2026 Feb 6;21(2):e0326000. doi: 10.1371/journal.pone.0326000 (PMC12880697; doi:10.1371/journal.pone.0326000)

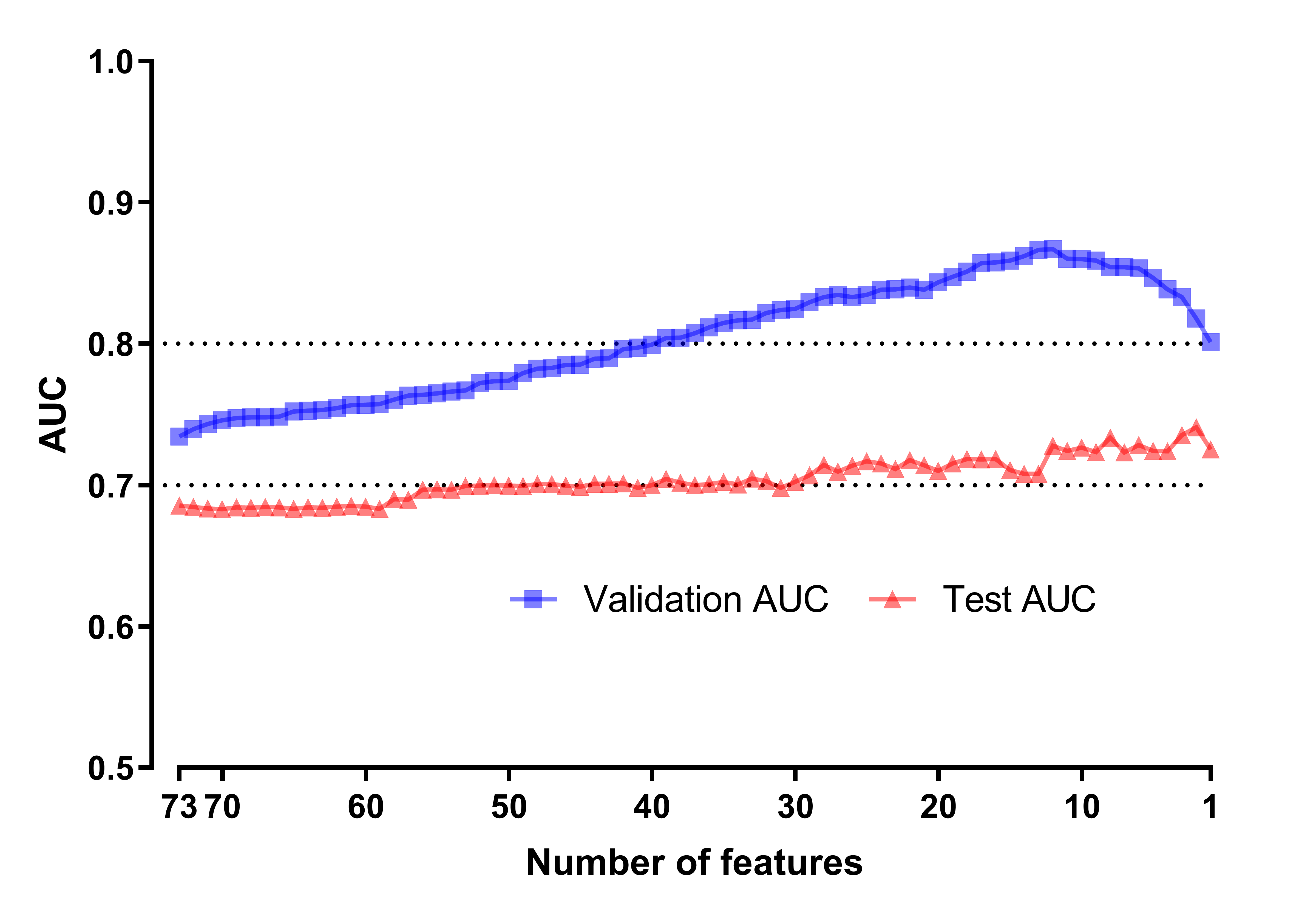

Supplement: S1 Fig — AUC at each step of backward stepwise feature selection for the binary logistic regression model, shown for one representative iteration (one of 100 repetitions). AUC, area under the receiver operating characteristic curve. (TIF) [file pone.0326000.s001.tif]

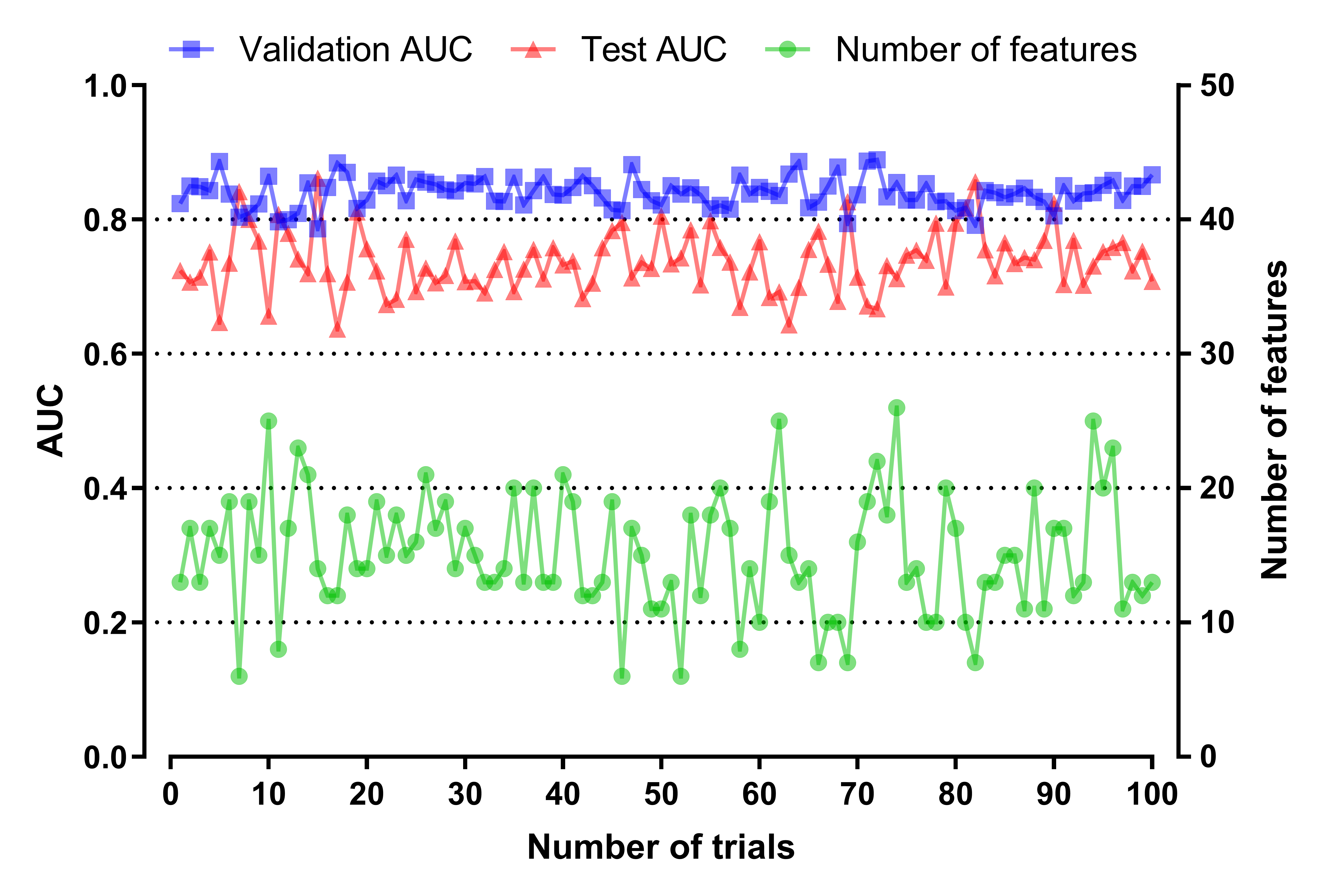

Supplement: S2 Fig — Model performance from backward feature selection repeated 100 times; the best-performing result is shown for each random split. AUC, area under the receiver operating characteristic curve. (TIF) [file pone.0326000.s002.tif]
