# Supplementary material for: Data-driven predictive modeling for massive intraoperative blood loss during living donor liver transplantation: Integrating machine learning techniques
Source: PLoS One. 2026 Feb 6;21(2):e0326000. doi: 10.1371/journal.pone.0326000 (PMC12880697; doi:10.1371/journal.pone.0326000)
Supplement: S1 Table — (DOCX) [file pone.0326000.s003.docx]

**Supplemental Table 1: List of abbreviations**

A2PI – Alpha-2-Plasmin Inhibitor

Alb - Albumin

ALF - Acute Liver Failure

ALP - Alkaline Phosphatase

ALT - Alanine Transaminase

AMY - Amylase

AnGap - Anion Gap

AST - Aspartate Transaminase

AT3 - Antithrombin III

BA - Biliary Atresia

BE - Base Excess

BH – Body Height

BS - Blood Sugar

BUN - Blood Urea Nitrogen

BW - Body Weight

Ca - Calcium

Ca2calc - Ionized Calcium

Che - Cholinesterase

Cl - Chloride

CPK - Creatine Phosphokinase

Crea - Creatinine

CRP - C-Reactive Protein

Dbil - Direct Bilirubin

Ddimer - D-Dimer

Ferri - Ferritin

Fib - Fibrinogen

GGT - Gamma-Glutamyl Transferase

Hb - Hemoglobin

HCO3 - Bicarbonate

Hct - Hematocrit

IBL - Intraoperative Bleeding

K - Potassium

Lactate - Lactate

LDH - Lactate Dehydrogenase

LL – Left Liver

LLS - Left Lateral Segment

LT - Liver Transplantation

MCH - Mean Corpuscular Hemoglobin

MCHC - Mean Corpuscular Hemoglobin Concentration

MCV - Mean Corpuscular Volume

Mg - Magnesium

Na - Sodium

NH3 - Ammonia

P - Phosphorus

PCO2 - Partial Pressure of Carbon Dioxide

PH - pH

PIC – Alpha-2-Plasmininhibitor-Plasmin Complex

Plasminogen - Plasminogen

Plt - Platelets

PO2 - Partial Pressure of Oxygen

ProteinC - Protein C

PTINR - Prothrombin Time International Normalized Ratio

PTpercent - Prothrombin Time Percent

RBC - Red Blood Cells

RL - Right Liver

RPL - Right Posterior Segment

TAT - Thrombin-Antithrombin Complex

TBA - Total Bile Acids

Tbil - Total Bilirubin

Tchol - Total Cholesterol

TCO2 - Total Carbon Dioxide

TG - Triglycerides

TP - Total Protein

UA - Uric Acid

VIF - Variance Inflation Factor

WBC - White Blood Cells
