# Supplementary material for: Data-driven predictive modeling for massive intraoperative blood loss during living donor liver transplantation: Integrating machine learning techniques
Source: PLoS One. 2026 Feb 6;21(2):e0326000. doi: 10.1371/journal.pone.0326000 (PMC12880697; doi:10.1371/journal.pone.0326000)
Supplement: S2 Table — (DOCX) [file pone.0326000.s004.docx]

**Supplemental Table 2: List of variance inflation factors**

| Variable | VIF |  | Variable | VIF |
| --- | --- | --- | --- | --- |
| A2PI | 5.97255 |  | LDH | 4.805717 |
| Alb | 3.700832 |  | MCH | 687.917472 |
| ALP | 4.314926 |  | MCHC | 92.39565 |
| ALT | 8.810128 |  | MCV | 552.390778 |
| AMY | 2.009802 |  | Mg | 1.933392 |
| AnGap | 2.383247 |  | Na | 8.436359 |
| AST | 10.312688 |  | NH3 | 1.946976 |
| AT3 | 6.79248 |  | P | 3.20923 |
| BE | 188.921528 |  | PCO2 | 59.463123 |
| BS | 1.641527 |  | PH | 43.805502 |
| BUN | 4.251717 |  | PIC | 3.167881 |
| Ca | 3.38077 |  | Plasminogen | 5.296453 |
| Ca2calc | 2.317197 |  | Plt | 2.419162 |
| Che | 3.456867 |  | PO2 | 1.598153 |
| Cl | 9.13698 |  | ProteinC | 7.20055 |
| CPK | 1.559591 |  | PTINR | 4.189638 |
| Crea | 5.268766 |  | PTpercent | 7.515981 |
| CRP | 2.715575 |  | RBC | 143.37964 |
| Dbil | 110.914508 |  | TAT | 1.937402 |
| Ddimer | 2.729546 |  | TBA | 3.984654 |
| Ferri | 3.698107 |  | Tbil | 111.528123 |
| Fib | 5.274548 |  | Tchol | 5.650772 |
| GGT | 4.290991 |  | TCO2 | 6946.386171 |
| Hb | 980.567795 |  | TG | 3.933022 |
| HCO3 | 6703.955531 |  | TP | 2.385535 |
| Hct | 1136.303372 |  | UA | 2.622419 |
| K | 3.800908 |  | WBC | 2.324854 |
| Lactate | 2.544913 |  |  |  |

VIF, variance inflation factor.

Abbreviations for laboratory test items are detailed in Supplemental Table 1.
