# Supplementary material for: Data-driven predictive modeling for massive intraoperative blood loss during living donor liver transplantation: Integrating machine learning techniques
Source: PLoS One. 2026 Feb 6;21(2):e0326000. doi: 10.1371/journal.pone.0326000 (PMC12880697; doi:10.1371/journal.pone.0326000)
Supplement: S3 Table — (DOCX) [file pone.0326000.s005.docx]

**Supplemental Table 3: Variable reduction using the backward stepwise method**

| **No. of Features** | **Validation AUC** | **Test AUC** | **Feature Names** |
| --- | --- | --- | --- |
| 73 | 0.734 | 0.685 | Indication_ALF, Indication_BA, Indication_graft failure, Indication_others, Gender, Age, BH, BW, Age_cat1, graft_LLS, graft_left, graft_mono, graft_post, graft_reducedLLS, graft_right, ABOi, rituximab, previous abdominal surgery, ALP, ALT, AMY, AnGap, AST, AT3, BE, Ca2calc, CPK, CRP, Ddimer, Fib, HCO3, PTINR, Lactate, LDH, MCH, MCHC, MCV, PCO2, PH, PIC, PO2, PTpercent, TAT, TCO2, A2PI, GGT, Alb, NH3, K, Ca, Crea, Cl, Che, TG, Na, Ferri, Plasminogen, ProteinC, Hct, Hb, Mg, Plt, BS, RBC, Tchol, Tbil, TBA, TP, Dbil, UA, BUN, WBC, P |
| 72 | 0.740 | 0.685 | Indication_ALF, Indication_BA, Indication_graft failure, Indication_others, Gender, Age, BH, BW, Age_cat1, graft_LLS, graft_left, graft_mono, graft_post, graft_reducedLLS, graft_right, ABOi, rituximab, previous abdominal surgery, ALP, ALT, AMY, AnGap, AST, AT3, BE, Ca2calc, CPK, CRP, Ddimer, Fib, HCO3, PTINR, Lactate, LDH, MCH, MCHC, MCV, PCO2, PH, PIC, PO2, PTpercent, TAT, TCO2, A2PI, GGT, Alb, NH3, K, Ca, Crea, Cl, Che, TG, Na, Ferri, ProteinC, Hct, Hb, Mg, Plt, BS, RBC, Tchol, Tbil, TBA, TP, Dbil, UA, BUN, WBC, P |
| 71 | 0.743 | 0.683 | Indication_ALF, Indication_BA, Indication_graft failure, Indication_others, Gender, Age, BH, BW, Age_cat1, graft_LLS, graft_left, graft_mono, graft_post, graft_reducedLLS, graft_right, ABOi, rituximab, previous abdominal surgery, ALP, ALT, AMY, AnGap, AST, AT3, BE, Ca2calc, CPK, CRP, Ddimer, Fib, HCO3, PTINR, Lactate, LDH, MCH, MCHC, MCV, PCO2, PH, PIC, PO2, PTpercent, TAT, TCO2, A2PI, GGT, Alb, NH3, K, Ca, Crea, Cl, Che, TG, Na, Ferri, ProteinC, Hct, Hb, Mg, Plt, BS, RBC, Tchol, Tbil, TBA, TP, Dbil, BUN, WBC, P |
| 70 | 0.746 | 0.683 | Indication_ALF, Indication_BA, Indication_graft failure, Indication_others, Gender, Age, BH, BW, Age_cat1, graft_LLS, graft_left, graft_mono, graft_post, graft_reducedLLS, graft_right, ABOi, rituximab, previous abdominal surgery, ALP, ALT, AMY, AnGap, AST, AT3, BE, CPK, CRP, Ddimer, Fib, HCO3, PTINR, Lactate, LDH, MCH, MCHC, MCV, PCO2, PH, PIC, PO2, PTpercent, TAT, TCO2, A2PI, GGT, Alb, NH3, K, Ca, Crea, Cl, Che, TG, Na, Ferri, ProteinC, Hct, Hb, Mg, Plt, BS, RBC, Tchol, Tbil, TBA, TP, Dbil, BUN, WBC, P |
| 69 | 0.747 | 0.684 | Indication_ALF, Indication_BA, Indication_graft failure, Indication_others, Gender, Age, BH, BW, Age_cat1, graft_LLS, graft_left, graft_mono, graft_post, graft_reducedLLS, graft_right, ABOi, rituximab, previous abdominal surgery, ALP, ALT, AMY, AnGap, AST, AT3, BE, CPK, CRP, Ddimer, Fib, HCO3, PTINR, Lactate, MCH, MCHC, MCV, PCO2, PH, PIC, PO2, PTpercent, TAT, TCO2, A2PI, GGT, Alb, NH3, K, Ca, Crea, Cl, Che, TG, Na, Ferri, ProteinC, Hct, Hb, Mg, Plt, BS, RBC, Tchol, Tbil, TBA, TP, Dbil, BUN, WBC, P |
| 68 | 0.748 | 0.684 | Indication_ALF, Indication_BA, Indication_graft failure, Indication_others, Gender, Age, BH, BW, Age_cat1, graft_LLS, graft_left, graft_mono, graft_post, graft_reducedLLS, graft_right, ABOi, rituximab, previous abdominal surgery, ALP, ALT, AMY, AnGap, AST, AT3, BE, CPK, CRP, Ddimer, Fib, HCO3, PTINR, Lactate, MCH, MCHC, MCV, PCO2, PH, PIC, PO2, PTpercent, TAT, A2PI, GGT, Alb, NH3, K, Ca, Crea, Cl, Che, TG, Na, Ferri, ProteinC, Hct, Hb, Mg, Plt, BS, RBC, Tchol, Tbil, TBA, TP, Dbil, BUN, WBC, P |
| 67 | 0.748 | 0.684 | Indication_ALF, Indication_BA, Indication_others, Gender, Age, BH, BW, Age_cat1, graft_LLS, graft_left, graft_mono, graft_post, graft_reducedLLS, graft_right, ABOi, rituximab, previous abdominal surgery, ALP, ALT, AMY, AnGap, AST, AT3, BE, CPK, CRP, Ddimer, Fib, HCO3, PTINR, Lactate, MCH, MCHC, MCV, PCO2, PH, PIC, PO2, PTpercent, TAT, A2PI, GGT, Alb, NH3, K, Ca, Crea, Cl, Che, TG, Na, Ferri, ProteinC, Hct, Hb, Mg, Plt, BS, RBC, Tchol, Tbil, TBA, TP, Dbil, BUN, WBC, P |
| 66 | 0.748 | 0.684 | Indication_ALF, Indication_BA, Indication_others, Gender, Age, BH, BW, Age_cat1, graft_LLS, graft_left, graft_mono, graft_post, graft_reducedLLS, graft_right, ABOi, rituximab, previous abdominal surgery, ALP, ALT, AMY, AnGap, AST, AT3, BE, CPK, CRP, Ddimer, Fib, HCO3, PTINR, Lactate, MCH, MCHC, MCV, PCO2, PIC, PO2, PTpercent, TAT, A2PI, GGT, Alb, NH3, K, Ca, Crea, Cl, Che, TG, Na, Ferri, ProteinC, Hct, Hb, Mg, Plt, BS, RBC, Tchol, Tbil, TBA, TP, Dbil, BUN, WBC, P |
| 65 | 0.752 | 0.683 | Indication_ALF, Indication_BA, Indication_others, Gender, Age, BH, BW, Age_cat1, graft_LLS, graft_left, graft_mono, graft_post, graft_reducedLLS, graft_right, ABOi, rituximab, previous abdominal surgery, ALP, ALT, AMY, AnGap, AST, AT3, BE, CPK, CRP, Ddimer, Fib, HCO3, PTINR, Lactate, MCH, MCHC, MCV, PCO2, PIC, PO2, PTpercent, TAT, A2PI, GGT, Alb, NH3, K, Ca, Crea, Cl, Che, TG, Na, Ferri, ProteinC, Hct, Hb, Mg, Plt, BS, RBC, Tchol, Tbil, TBA, TP, Dbil, WBC, P |
| 64 | 0.753 | 0.684 | Indication_ALF, Indication_BA, Gender, Age, BH, BW, Age_cat1, graft_LLS, graft_left, graft_mono, graft_post, graft_reducedLLS, graft_right, ABOi, rituximab, previous abdominal surgery, ALP, ALT, AMY, AnGap, AST, AT3, BE, CPK, CRP, Ddimer, Fib, HCO3, PTINR, Lactate, MCH, MCHC, MCV, PCO2, PIC, PO2, PTpercent, TAT, A2PI, GGT, Alb, NH3, K, Ca, Crea, Cl, Che, TG, Na, Ferri, ProteinC, Hct, Hb, Mg, Plt, BS, RBC, Tchol, Tbil, TBA, TP, Dbil, WBC, P |
| 63 | 0.753 | 0.684 | Indication_ALF, Indication_BA, Gender, Age, BH, BW, Age_cat1, graft_LLS, graft_left, graft_mono, graft_post, graft_reducedLLS, graft_right, ABOi, rituximab, previous abdominal surgery, ALP, ALT, AMY, AnGap, AST, AT3, CPK, CRP, Ddimer, Fib, HCO3, PTINR, Lactate, MCH, MCHC, MCV, PCO2, PIC, PO2, PTpercent, TAT, A2PI, GGT, Alb, NH3, K, Ca, Crea, Cl, Che, TG, Na, Ferri, ProteinC, Hct, Hb, Mg, Plt, BS, RBC, Tchol, Tbil, TBA, TP, Dbil, WBC, P |
| 62 | 0.755 | 0.685 | Indication_ALF, Indication_BA, Gender, Age, BH, BW, Age_cat1, graft_LLS, graft_left, graft_mono, graft_post, graft_reducedLLS, graft_right, ABOi, rituximab, previous abdominal surgery, ALP, ALT, AMY, AnGap, AST, AT3, CPK, CRP, Ddimer, Fib, PTINR, Lactate, MCH, MCHC, MCV, PCO2, PIC, PO2, PTpercent, TAT, A2PI, GGT, Alb, NH3, K, Ca, Crea, Cl, Che, TG, Na, Ferri, ProteinC, Hct, Hb, Mg, Plt, BS, RBC, Tchol, Tbil, TBA, TP, Dbil, WBC, P |
| 61 | 0.757 | 0.685 | Indication_BA, Gender, Age, BH, BW, Age_cat1, graft_LLS, graft_left, graft_mono, graft_post, graft_reducedLLS, graft_right, ABOi, rituximab, previous abdominal surgery, ALP, ALT, AMY, AnGap, AST, AT3, CPK, CRP, Ddimer, Fib, PTINR, Lactate, MCH, MCHC, MCV, PCO2, PIC, PO2, PTpercent, TAT, A2PI, GGT, Alb, NH3, K, Ca, Crea, Cl, Che, TG, Na, Ferri, ProteinC, Hct, Hb, Mg, Plt, BS, RBC, Tchol, Tbil, TBA, TP, Dbil, WBC, P |
| 60 | 0.757 | 0.685 | Indication_BA, Gender, Age, BH, BW, Age_cat1, graft_LLS, graft_left, graft_mono, graft_post, graft_reducedLLS, graft_right, ABOi, rituximab, previous abdominal surgery, ALP, ALT, AMY, AnGap, AST, AT3, CPK, CRP, Ddimer, Fib, PTINR, Lactate, MCH, MCHC, MCV, PCO2, PIC, PO2, PTpercent, TAT, A2PI, GGT, Alb, NH3, K, Ca, Crea, Cl, Che, TG, Na, Ferri, ProteinC, Hct, Hb, Mg, Plt, BS, Tchol, Tbil, TBA, TP, Dbil, WBC, P |
| 59 | 0.757 | 0.683 | Indication_BA, Gender, Age, BH, BW, Age_cat1, graft_LLS, graft_left, graft_mono, graft_post, graft_reducedLLS, graft_right, ABOi, rituximab, previous abdominal surgery, ALP, ALT, AMY, AnGap, AST, AT3, CPK, CRP, Ddimer, Fib, PTINR, Lactate, MCH, MCV, PCO2, PIC, PO2, PTpercent, TAT, A2PI, GGT, Alb, NH3, K, Ca, Crea, Cl, Che, TG, Na, Ferri, ProteinC, Hct, Hb, Mg, Plt, BS, Tchol, Tbil, TBA, TP, Dbil, WBC, P |
| 58 | 0.760 | 0.690 | Indication_BA, Gender, Age, BH, BW, Age_cat1, graft_LLS, graft_left, graft_mono, graft_post, graft_reducedLLS, graft_right, ABOi, rituximab, previous abdominal surgery, ALP, ALT, AMY, AnGap, AST, AT3, CPK, CRP, Ddimer, Fib, PTINR, Lactate, MCH, MCV, PIC, PO2, PTpercent, TAT, A2PI, GGT, Alb, NH3, K, Ca, Crea, Cl, Che, TG, Na, Ferri, ProteinC, Hct, Hb, Mg, Plt, BS, Tchol, Tbil, TBA, TP, Dbil, WBC, P |
| 57 | 0.763 | 0.690 | Indication_BA, Gender, Age, BH, BW, Age_cat1, graft_LLS, graft_left, graft_mono, graft_post, graft_reducedLLS, graft_right, ABOi, rituximab, previous abdominal surgery, ALP, ALT, AMY, AnGap, AST, AT3, CPK, CRP, Ddimer, Fib, PTINR, Lactate, MCH, MCV, PIC, PO2, PTpercent, TAT, A2PI, GGT, Alb, NH3, K, Ca, Crea, Cl, Che, TG, Na, Ferri, Hct, Hb, Mg, Plt, BS, Tchol, Tbil, TBA, TP, Dbil, WBC, P |
| 56 | 0.764 | 0.697 | Indication_BA, Gender, Age, BH, BW, Age_cat1, graft_LLS, graft_left, graft_mono, graft_post, graft_reducedLLS, graft_right, ABOi, rituximab, previous abdominal surgery, ALP, ALT, AMY, AnGap, AST, AT3, CPK, CRP, Ddimer, Fib, Lactate, MCH, MCV, PIC, PO2, PTpercent, TAT, A2PI, GGT, Alb, NH3, K, Ca, Crea, Cl, Che, TG, Na, Ferri, Hct, Hb, Mg, Plt, BS, Tchol, Tbil, TBA, TP, Dbil, WBC, P |
| 55 | 0.765 | 0.697 | Indication_BA, Gender, Age, BH, BW, Age_cat1, graft_LLS, graft_left, graft_mono, graft_post, graft_reducedLLS, graft_right, ABOi, rituximab, previous abdominal surgery, ALP, ALT, AMY, AnGap, AST, AT3, CPK, CRP, Ddimer, Fib, Lactate, MCH, MCV, PIC, PO2, PTpercent, TAT, GGT, Alb, NH3, K, Ca, Crea, Cl, Che, TG, Na, Ferri, Hct, Hb, Mg, Plt, BS, Tchol, Tbil, TBA, TP, Dbil, WBC, P |
| 54 | 0.766 | 0.697 | Indication_BA, Gender, Age, BH, BW, Age_cat1, graft_LLS, graft_left, graft_mono, graft_post, graft_reducedLLS, graft_right, ABOi, rituximab, previous abdominal surgery, ALP, ALT, AMY, AnGap, AST, AT3, CPK, CRP, Ddimer, Fib, Lactate, MCH, MCV, PIC, PO2, PTpercent, TAT, GGT, Alb, NH3, K, Ca, Crea, Cl, Che, TG, Na, Ferri, Hct, Hb, Mg, Plt, BS, Tchol, Tbil, TBA, TP, WBC, P |
| 53 | 0.767 | 0.699 | Indication_BA, Gender, Age, BH, BW, Age_cat1, graft_LLS, graft_left, graft_mono, graft_post, graft_reducedLLS, graft_right, ABOi, rituximab, previous abdominal surgery, ALP, ALT, AMY, AnGap, AST, AT3, CPK, CRP, Ddimer, Fib, Lactate, MCH, MCV, PIC, PO2, PTpercent, TAT, GGT, Alb, NH3, K, Ca, Crea, Cl, Che, TG, Na, Ferri, Hct, Hb, Mg, Plt, BS, Tchol, Tbil, TBA, TP, P |
| 52 | 0.772 | 0.699 | Indication_BA, Gender, Age, BH, BW, Age_cat1, graft_LLS, graft_left, graft_mono, graft_post, graft_reducedLLS, graft_right, ABOi, rituximab, previous abdominal surgery, ALP, ALT, AMY, AST, AT3, CPK, CRP, Ddimer, Fib, Lactate, MCH, MCV, PIC, PO2, PTpercent, TAT, GGT, Alb, NH3, K, Ca, Crea, Cl, Che, TG, Na, Ferri, Hct, Hb, Mg, Plt, BS, Tchol, Tbil, TBA, TP, P |
| 51 | 0.773 | 0.700 | Indication_BA, Gender, Age, BH, BW, graft_LLS, graft_left, graft_mono, graft_post, graft_reducedLLS, graft_right, ABOi, rituximab, previous abdominal surgery, ALP, ALT, AMY, AST, AT3, CPK, CRP, Ddimer, Fib, Lactate, MCH, MCV, PIC, PO2, PTpercent, TAT, GGT, Alb, NH3, K, Ca, Crea, Cl, Che, TG, Na, Ferri, Hct, Hb, Mg, Plt, BS, Tchol, Tbil, TBA, TP, P |
| 50 | 0.774 | 0.700 | Indication_BA, Gender, Age, BW, graft_LLS, graft_left, graft_mono, graft_post, graft_reducedLLS, graft_right, ABOi, rituximab, previous abdominal surgery, ALP, ALT, AMY, AST, AT3, CPK, CRP, Ddimer, Fib, Lactate, MCH, MCV, PIC, PO2, PTpercent, TAT, GGT, Alb, NH3, K, Ca, Crea, Cl, Che, TG, Na, Ferri, Hct, Hb, Mg, Plt, BS, Tchol, Tbil, TBA, TP, P |
| 49 | 0.779 | 0.699 | Indication_BA, Gender, Age, BW, graft_LLS, graft_left, graft_mono, graft_post, graft_reducedLLS, graft_right, rituximab, previous abdominal surgery, ALP, ALT, AMY, AST, AT3, CPK, CRP, Ddimer, Fib, Lactate, MCH, MCV, PIC, PO2, PTpercent, TAT, GGT, Alb, NH3, K, Ca, Crea, Cl, Che, TG, Na, Ferri, Hct, Hb, Mg, Plt, BS, Tchol, Tbil, TBA, TP, P |
| 48 | 0.782 | 0.701 | Indication_BA, Gender, Age, BW, graft_LLS, graft_left, graft_mono, graft_post, graft_reducedLLS, graft_right, previous abdominal surgery, ALP, ALT, AMY, AST, AT3, CPK, CRP, Ddimer, Fib, Lactate, MCH, MCV, PIC, PO2, PTpercent, TAT, GGT, Alb, NH3, K, Ca, Crea, Cl, Che, TG, Na, Ferri, Hct, Hb, Mg, Plt, BS, Tchol, Tbil, TBA, TP, P |
| 47 | 0.783 | 0.701 | Indication_BA, Gender, Age, BW, graft_LLS, graft_left, graft_mono, graft_post, graft_reducedLLS, previous abdominal surgery, ALP, ALT, AMY, AST, AT3, CPK, CRP, Ddimer, Fib, Lactate, MCH, MCV, PIC, PO2, PTpercent, TAT, GGT, Alb, NH3, K, Ca, Crea, Cl, Che, TG, Na, Ferri, Hct, Hb, Mg, Plt, BS, Tchol, Tbil, TBA, TP, P |
| 46 | 0.785 | 0.700 | Indication_BA, Gender, Age, BW, graft_LLS, graft_mono, graft_post, graft_reducedLLS, previous abdominal surgery, ALP, ALT, AMY, AST, AT3, CPK, CRP, Ddimer, Fib, Lactate, MCH, MCV, PIC, PO2, PTpercent, TAT, GGT, Alb, NH3, K, Ca, Crea, Cl, Che, TG, Na, Ferri, Hct, Hb, Mg, Plt, BS, Tchol, Tbil, TBA, TP, P |
| 45 | 0.785 | 0.699 | Indication_BA, Gender, Age, BW, graft_LLS, graft_mono, graft_post, graft_reducedLLS, previous abdominal surgery, ALP, ALT, AMY, AST, AT3, CPK, CRP, Ddimer, Fib, Lactate, MCH, MCV, PIC, PO2, PTpercent, TAT, GGT, Alb, NH3, K, Ca, Crea, Cl, Che, TG, Na, Ferri, Hct, Mg, Plt, BS, Tchol, Tbil, TBA, TP, P |
| 44 | 0.789 | 0.701 | Indication_BA, Gender, Age, BW, graft_LLS, graft_mono, graft_reducedLLS, previous abdominal surgery, ALP, ALT, AMY, AST, AT3, CPK, CRP, Ddimer, Fib, Lactate, MCH, MCV, PIC, PO2, PTpercent, TAT, GGT, Alb, NH3, K, Ca, Crea, Cl, Che, TG, Na, Ferri, Hct, Mg, Plt, BS, Tchol, Tbil, TBA, TP, P |
| 43 | 0.790 | 0.701 | Indication_BA, Gender, Age, BW, graft_LLS, graft_mono, graft_reducedLLS, previous abdominal surgery, ALP, ALT, AMY, AST, AT3, CPK, CRP, Ddimer, Fib, Lactate, MCV, PIC, PO2, PTpercent, TAT, GGT, Alb, NH3, K, Ca, Crea, Cl, Che, TG, Na, Ferri, Hct, Mg, Plt, BS, Tchol, Tbil, TBA, TP, P |
| 42 | 0.796 | 0.701 | Indication_BA, Gender, Age, BW, graft_LLS, graft_mono, graft_reducedLLS, previous abdominal surgery, ALP, ALT, AMY, AST, AT3, CPK, CRP, Ddimer, Fib, Lactate, MCV, PIC, PO2, PTpercent, GGT, Alb, NH3, K, Ca, Crea, Cl, Che, TG, Na, Ferri, Hct, Mg, Plt, BS, Tchol, Tbil, TBA, TP, P |
| 41 | 0.797 | 0.698 | Indication_BA, Gender, Age, BW, graft_LLS, graft_mono, graft_reducedLLS, previous abdominal surgery, ALP, ALT, AMY, AST, AT3, CPK, CRP, Ddimer, Fib, Lactate, MCV, PIC, PO2, GGT, Alb, NH3, K, Ca, Crea, Cl, Che, TG, Na, Ferri, Hct, Mg, Plt, BS, Tchol, Tbil, TBA, TP, P |
| 40 | 0.799 | 0.700 | Indication_BA, Gender, Age, graft_LLS, graft_mono, graft_reducedLLS, previous abdominal surgery, ALP, ALT, AMY, AST, AT3, CPK, CRP, Ddimer, Fib, Lactate, MCV, PIC, PO2, GGT, Alb, NH3, K, Ca, Crea, Cl, Che, TG, Na, Ferri, Hct, Mg, Plt, BS, Tchol, Tbil, TBA, TP, P |
| 39 | 0.804 | 0.704 | Indication_BA, Gender, Age, graft_LLS, graft_mono, graft_reducedLLS, previous abdominal surgery, ALP, ALT, AMY, AST, AT3, CPK, CRP, Ddimer, Fib, Lactate, MCV, PIC, GGT, Alb, NH3, K, Ca, Crea, Cl, Che, TG, Na, Ferri, Hct, Mg, Plt, BS, Tchol, Tbil, TBA, TP, P |
| 38 | 0.804 | 0.702 | Indication_BA, Gender, Age, graft_LLS, graft_mono, graft_reducedLLS, previous abdominal surgery, ALP, ALT, AMY, AST, AT3, CPK, CRP, Ddimer, Fib, MCV, PIC, GGT, Alb, NH3, K, Ca, Crea, Cl, Che, TG, Na, Ferri, Hct, Mg, Plt, BS, Tchol, Tbil, TBA, TP, P |
| 37 | 0.807 | 0.700 | Indication_BA, Gender, Age, graft_LLS, graft_mono, graft_reducedLLS, previous abdominal surgery, ALP, ALT, AMY, AST, AT3, CPK, CRP, Ddimer, Fib, MCV, PIC, GGT, Alb, NH3, K, Ca, Crea, Che, TG, Na, Ferri, Hct, Mg, Plt, BS, Tchol, Tbil, TBA, TP, P |
| 36 | 0.812 | 0.701 | Gender, Age, graft_LLS, graft_mono, graft_reducedLLS, previous abdominal surgery, ALP, ALT, AMY, AST, AT3, CPK, CRP, Ddimer, Fib, MCV, PIC, GGT, Alb, NH3, K, Ca, Crea, Che, TG, Na, Ferri, Hct, Mg, Plt, BS, Tchol, Tbil, TBA, TP, P |
| 35 | 0.815 | 0.702 | Gender, Age, graft_LLS, graft_mono, graft_reducedLLS, previous abdominal surgery, ALP, ALT, AMY, AST, AT3, CPK, CRP, Ddimer, Fib, MCV, PIC, GGT, Alb, NH3, K, Ca, Crea, Che, TG, Na, Ferri, Hct, Mg, Plt, BS, Tchol, Tbil, TBA, P |
| 34 | 0.817 | 0.700 | Gender, Age, graft_LLS, graft_mono, graft_reducedLLS, previous abdominal surgery, ALP, ALT, AMY, AST, AT3, CPK, CRP, Ddimer, Fib, MCV, GGT, Alb, NH3, K, Ca, Crea, Che, TG, Na, Ferri, Hct, Mg, Plt, BS, Tchol, Tbil, TBA, P |
| 33 | 0.817 | 0.705 | Gender, Age, graft_LLS, graft_mono, graft_reducedLLS, previous abdominal surgery, ALP, ALT, AMY, AST, AT3, CPK, CRP, Ddimer, Fib, MCV, GGT, Alb, NH3, K, Ca, Crea, Che, TG, Na, Ferri, Hct, Plt, BS, Tchol, Tbil, TBA, P |
| 32 | 0.822 | 0.703 | Gender, Age, graft_LLS, graft_mono, graft_reducedLLS, previous abdominal surgery, ALP, ALT, AMY, AST, AT3, CPK, CRP, Ddimer, Fib, MCV, GGT, Alb, NH3, Ca, Crea, Che, TG, Na, Ferri, Hct, Plt, BS, Tchol, Tbil, TBA, P |
| 31 | 0.824 | 0.698 | Gender, graft_LLS, graft_mono, graft_reducedLLS, previous abdominal surgery, ALP, ALT, AMY, AST, AT3, CPK, CRP, Ddimer, Fib, MCV, GGT, Alb, NH3, Ca, Crea, Che, TG, Na, Ferri, Hct, Plt, BS, Tchol, Tbil, TBA, P |
| 30 | 0.825 | 0.702 | Gender, graft_LLS, graft_mono, graft_reducedLLS, previous abdominal surgery, ALP, ALT, AMY, AT3, CPK, CRP, Ddimer, Fib, MCV, GGT, Alb, NH3, Ca, Crea, Che, TG, Na, Ferri, Hct, Plt, BS, Tchol, Tbil, TBA, P |
| 29 | 0.829 | 0.707 | Gender, graft_LLS, graft_mono, graft_reducedLLS, previous abdominal surgery, ALP, AMY, AT3, CPK, CRP, Ddimer, Fib, MCV, GGT, Alb, NH3, Ca, Crea, Che, TG, Na, Ferri, Hct, Plt, BS, Tchol, Tbil, TBA, P |
| 28 | 0.833 | 0.714 | Gender, graft_LLS, graft_mono, graft_reducedLLS, previous abdominal surgery, ALP, AMY, AT3, CPK, CRP, Ddimer, Fib, MCV, GGT, Alb, NH3, Ca, Crea, Che, Na, Ferri, Hct, Plt, BS, Tchol, Tbil, TBA, P |
| 27 | 0.835 | 0.710 | Gender, graft_LLS, graft_mono, graft_reducedLLS, previous abdominal surgery, ALP, AMY, AT3, CPK, CRP, Ddimer, Fib, MCV, GGT, Alb, NH3, Ca, Crea, Che, Na, Ferri, Hct, BS, Tchol, Tbil, TBA, P |
| 26 | 0.833 | 0.714 | Gender, graft_LLS, graft_mono, graft_reducedLLS, previous abdominal surgery, ALP, AMY, AT3, CPK, CRP, Ddimer, Fib, GGT, Alb, NH3, Ca, Crea, Che, Na, Ferri, Hct, BS, Tchol, Tbil, TBA, P |
| 25 | 0.835 | 0.717 | Gender, graft_LLS, graft_reducedLLS, previous abdominal surgery, ALP, AMY, AT3, CPK, CRP, Ddimer, Fib, GGT, Alb, NH3, Ca, Crea, Che, Na, Ferri, Hct, BS, Tchol, Tbil, TBA, P |
| 24 | 0.838 | 0.715 | Gender, graft_LLS, graft_reducedLLS, previous abdominal surgery, ALP, AMY, AT3, CPK, CRP, Ddimer, Fib, GGT, Alb, NH3, Crea, Che, Na, Ferri, Hct, BS, Tchol, Tbil, TBA, P |
| 23 | 0.838 | 0.711 | Gender, graft_LLS, graft_reducedLLS, ALP, AMY, AT3, CPK, CRP, Ddimer, Fib, GGT, Alb, NH3, Crea, Che, Na, Ferri, Hct, BS, Tchol, Tbil, TBA, P |
| 22 | 0.840 | 0.718 | Gender, graft_LLS, graft_reducedLLS, ALP, AMY, AT3, CPK, CRP, Ddimer, Fib, GGT, Alb, NH3, Crea, Che, Na, Ferri, Hct, BS, Tchol, Tbil, TBA |
| 21 | 0.838 | 0.714 | Gender, graft_LLS, graft_reducedLLS, ALP, AMY, AT3, CPK, CRP, Ddimer, Fib, GGT, Alb, NH3, Crea, Na, Ferri, Hct, BS, Tchol, Tbil, TBA |
| 20 | 0.844 | 0.710 | Gender, graft_LLS, graft_reducedLLS, ALP, AMY, AT3, CPK, CRP, Ddimer, Fib, GGT, NH3, Crea, Na, Ferri, Hct, BS, Tchol, Tbil, TBA |
| 19 | 0.847 | 0.715 | Gender, graft_LLS, graft_reducedLLS, ALP, AMY, AT3, CPK, CRP, Ddimer, Fib, GGT, NH3, Crea, Na, Ferri, Hct, Tchol, Tbil, TBA |
| 18 | 0.851 | 0.718 | Gender, graft_LLS, graft_reducedLLS, ALP, AMY, AT3, CPK, CRP, Ddimer, Fib, GGT, NH3, Crea, Ferri, Hct, Tchol, Tbil, TBA |
| 17 | 0.857 | 0.718 | Gender, graft_LLS, graft_reducedLLS, ALP, AMY, AT3, CPK, CRP, Ddimer, Fib, GGT, NH3, Crea, Hct, Tchol, Tbil, TBA |
| 16 | 0.857 | 0.718 | Gender, graft_LLS, graft_reducedLLS, ALP, AMY, AT3, CPK, CRP, Ddimer, Fib, GGT, NH3, Crea, Hct, Tbil, TBA |
| 15 | 0.859 | 0.710 | Gender, graft_LLS, graft_reducedLLS, ALP, AT3, CPK, CRP, Ddimer, Fib, GGT, NH3, Crea, Hct, Tbil, TBA |
| 14 | 0.862 | 0.708 | Gender, graft_LLS, graft_reducedLLS, AT3, CPK, CRP, Ddimer, Fib, GGT, NH3, Crea, Hct, Tbil, TBA |
| 13 | 0.867 | 0.708 | Gender, graft_LLS, graft_reducedLLS, AT3, CPK, CRP, Ddimer, Fib, GGT, NH3, Crea, Hct, Tbil |
| 12 | 0.867 | 0.728 | graft_LLS, graft_reducedLLS, AT3, CPK, CRP, Ddimer, Fib, GGT, NH3, Crea, Hct, Tbil |
| 11 | 0.860 | 0.724 | graft_LLS, graft_reducedLLS, AT3, CPK, CRP, Ddimer, Fib, GGT, Crea, Hct, Tbil |
| 10 | 0.860 | 0.726 | graft_LLS, graft_reducedLLS, AT3, CPK, Ddimer, Fib, GGT, Crea, Hct, Tbil |
| 9 | 0.859 | 0.723 | graft_LLS, graft_reducedLLS, AT3, CPK, Ddimer, GGT, Crea, Hct, Tbil |
| 8 | 0.854 | 0.734 | graft_LLS, graft_reducedLLS, AT3, Ddimer, GGT, Crea, Hct, Tbil |
| 7 | 0.854 | 0.723 | graft_reducedLLS, AT3, Ddimer, GGT, Crea, Hct, Tbil |
| 6 | 0.853 | 0.728 | AT3, Ddimer, GGT, Crea, Hct, Tbil |
| 5 | 0.847 | 0.724 | AT3, Ddimer, GGT, Crea, Tbil |
| 4 | 0.839 | 0.724 | AT3, Ddimer, Crea, Tbil |
| 3 | 0.833 | 0.735 | AT3, Ddimer, Tbil |
| 2 | 0.818 | 0.741 | AT3, Ddimer |
| 1 | 0.801 | 0.725 | AT3 |

Abbreviations for laboratory test items are detailed in Supplemental Table 1.
