# Supplementary material for: Data-driven predictive modeling for massive intraoperative blood loss during living donor liver transplantation: Integrating machine learning techniques
Source: PLoS One. 2026 Feb 6;21(2):e0326000. doi: 10.1371/journal.pone.0326000 (PMC12880697; doi:10.1371/journal.pone.0326000)
Supplement: S4 Table — (DOCX) [file pone.0326000.s006.docx]

**Supplemental Table 4: Performance in OLT cases**

| Case No. | Calicurated risk (%) | IBL (mL) | aIBL (mL/kg) | Correctly classified |
| --- | --- | --- | --- | --- |
| 1 | 11.9 | 2150 | 289.6 | NO |
| 2 | 97.2 | 12360 | 643.8 | YES |
| 3 | 49.6 | 31680 | 945.7 | NO |
| 4 | 5.4 | 550 | 40.7 | YES |
| 5 | 69.7 | 1115 | 38.1 | NO |
| 6 | 96.7 | 8990 | 160.2 | YES |
| 7 | 13.3 | 780 | 37.9 | YES |
| 8 | 3.1 | 650 | 15.4 | YES |
| 9 | 97.5 | 9250 | 153.9 | YES |
| 10 | 97.2 | 8960 | 171.3 | YES |
| 11 | 98.6 | 8200 | 132.5 | YES |
| 12 | 98.8 | 8580 | 92.3 | YES |
| 13 | 94.7 | 1305 | 25.0 | NO |

IBL, intraoperative bleeding; OLT, orthotopic liver transplantation

Predicted event was defined as Risk ≥ 50 percent. Observed event was defined as aIBL ≥ 80 mL/kg. Correctly classified indicates agreement between the predicted class and the observed outcome.
