# Supplementary material for: Data-driven predictive modeling for massive intraoperative blood loss during living donor liver transplantation: Integrating machine learning techniques
Source: PLoS One. 2026 Feb 6;21(2):e0326000. doi: 10.1371/journal.pone.0326000 (PMC12880697; doi:10.1371/journal.pone.0326000)
Supplement: S1 File — (DOCX) [file pone.0326000.s007.docx]

**Supplementary Methods**

***Backward feature selection***

The backward stepwise selection algorithm was applied to develop a logistic regression model for feature selection. The process began with a full model incorporating all available features, using repeated stratified 3-fold cross-validation to ensure robust evaluation. Each fold was scaled individually to standardize feature values, maintaining consistency during model training and testing. At each iteration, the logistic regression model's coefficients were computed for the selected subset of features across all folds. The mean coefficients and intercept were then calculated, and the area under the receiver operating characteristic curve (AUC) was evaluated for both the validation and test datasets. The features were ranked according to the absolute values of their coefficients, and the least influential feature, defined as the one with the smallest absolute coefficient, was removed from the subset. This iterative process continued until only a single feature remained. To determine the optimal feature set, the model's performance was assessed at each step using the mean validation AUC, calculated over ten repetitions of three-fold cross-validation. The subset yielding the highest validation AUC was selected as the optimal feature set. Finally, the model's predictive performance was then evaluated on an independent test dataset, where key metrics, including the test AUC, were computed to ensure model reliability. The selected features and their corresponding coefficients were saved for further analysis. To enhance the robustness of the feature selection process, the entire process was repeated 100 times, each using a distinct random split of the data. The algorithm was implemented utilizing Python libraries, including Scikit-learn for machine learning workflows and Pandas for efficient data manipulation.^1^

***Development of an online calculator for estimating the risk of massive aIBL***

Based on the results from the backward stepwise selection process, we developed an online calculator to estimate the probability of massive aIBL. To assess the consistency of selected features across multiple random data splits, index stability was evaluated using the occurrence rate and absolute mean weight. The occurrence rate was defined as the proportion of iterations in which a given feature was retained as a predictive factor during the backward stepwise selection process. The absolute mean weight was calculated as the average absolute value of the logistic regression coefficients for each feature across all iterations and was used to quantify the predictive impact of each feature. Features demonstrating both a high occurrence rate and a large absolute mean weight were considered robust predictors and were incorporated into the final logistic model. To ensure model reliability, features were selected based on the following criteria: an occurrence rate of at least 50% and an absolute mean weight of 0.5 or greater.

The selected features were then standardized to ensure consistency in input values before applying their respective weights in the final model. The standardization process involved transforming each feature based on its mean and standard deviation. A weighted sum of these standardized values was computed using the logistic regression coefficients obtained from model fitting. Finally, the probability of massive aIBL was estimated using a logistic function, which converted the weighted sum into a probability value ranging from 0 to 1.

**References**

1. Pedregosa F. Scikit-learn : Machine learning in python. *J Machine Learn Res.* 2011;12:2825-2830.
